# Siponimod vs placebo in active secondary progressive multiple sclerosis: a post hoc analysis from the phase 3 EXPAND study

**DOI:** 10.1007/s00415-022-11166-z

**Published:** 2022-05-31

**Authors:** Ralf Gold, Daniela Piani-Meier, Ludwig Kappos, Amit Bar-Or, Patrick Vermersch, Gavin Giovannoni, Robert J. Fox, Douglas L. Arnold, Ralph H. B. Benedict, Iris-Katharina Penner, Nicolas Rouyrre, Ajay Kilaru, Göril Karlsson, Shannon Ritter, Frank Dahlke, Thomas Hach, Bruce A. C. Cree

**Affiliations:** 1 Department of Neurology, St. Josef Hospital and Ruhr University of Bochum, Bochum, Germany; 2 Novartis Pharma AG, Basel, Switzerland; 3 Research Center for Clinical Neuroimmunology and Neuroscience Basel (RC2NB) and Multiple Sclerosis Center, Departments of Head, Spine and Neuromedicine, Clinical Research, Biomedicine, and Biomedical Engineering, University Hospital, University of Basel, Basel, Switzerland; 4 Center for Neuroinflammation and Experimental Therapeutics, and Department of Neurology, Perelman School of Medicine, University of Pennsylvania, Philadelphia, PA USA; 5 University of Lille, Inserm U1172 LilNCog, CHU Lille, FHU Precise, Lille, France; 6 Blizard Institute, Barts and The London School of Medicine and Dentistry, Queen Mary University of London, London, UK; 7 Mellen Center for Multiple Sclerosis Treatment and Research, Neurological Institute, Cleveland Clinic, Cleveland, OH USA; 8 NeuroRx Research, Montreal, QC, Canada and Montreal Neurological Institute, McGill University, Montreal, QC Canada; 9 Department of Neurology, University at Buffalo, Buffalo, NY USA; 10 Department of Neurology, Inselspital, Bern University Hospital, University of Bern, Bern, Switzerland; 11 UCSF Weill Institute for Neurosciences, Department of Neurology, University of California, San Francisco, San Francisco, CA USA

**Keywords:** Siponimod, EXPAND, Active secondary progressive multiple sclerosis, Disability progression, MRI, Cognition

## Abstract

**Background:**

Siponimod is a sphingosine 1-phosphate receptor modulator approved for active secondary progressive multiple sclerosis (aSPMS) in most countries; however, phase 3 EXPAND study data are from an SPMS population with/without disease activity. A need exists to characterize efficacy/safety of siponimod in aSPMS.

**Methods:**

Post hoc analysis of participants with aSPMS (≥ 1 relapse in 2 years before study and/or ≥ 1 T1 gadolinium-enhancing [Gd +] magnetic resonance imaging [MRI] lesions at baseline) receiving oral siponimod (2 mg/day) or placebo for up to 3 years in EXPAND. Endpoints: 3-month/6-month confirmed disability progression (3mCDP/6mCDP); 3-month confirmed ≥ 20% worsening in Timed 25-Foot Walk (T25FW); 6-month confirmed improvement/worsening in Symbol Digit Modalities Test (SDMT) scores (≥ 4-point change); T2 lesion volume (T2LV) change from baseline; number of T1 Gd + lesions baseline–month 24; number of new/enlarging (N/E) T2 lesions over all visits.

**Results:**

Data from 779 participants with aSPMS were analysed. Siponimod reduced risk of 3mCDP/6mCDP vs placebo (by 31%/37%, respectively; *p* < 0.01); there was no significant effect on T25FW. Siponimod increased likelihood of 6-month confirmed SDMT improvement vs placebo (by 62%; *p* = 0.007) and reduced risk of 6-month confirmed SDMT worsening (by 27%; *p* = 0.060). Siponimod was associated with less increase in T2LV (1316.3 vs 13.3 mm^3^; *p* < 0.0001), and fewer T1 Gd + and N/E T2 lesions than placebo (85% and 80% reductions, respectively; *p* < 0.0001).

**Conclusions:**

In aSPMS, siponimod reduced risk of disability progression and was associated with benefits on cognition and MRI outcomes vs placebo.

**Trial registration:**

*ClinicalTrials.gov number*: NCT01665144.

**Supplementary Information:**

The online version contains supplementary material available at 10.1007/s00415-022-11166-z.

## Introduction

In most patients, multiple sclerosis (MS) begins with a relapsing–remitting course, in which relapses are followed by periods of remission [1]. Relapsing–remitting MS (RRMS) is often followed by a stage of worsening neurological function that occurs independently of relapse [2, 3], known as secondary progressive MS (SPMS). SPMS is considered active if there is recent evidence of clinical relapses and/or magnetic resonance imaging (MRI) lesion activity [3].

SPMS is associated with progressive accumulation of physical disability, as defined by the Expanded Disability Status Scale (EDSS), which may be evident in patients with EDSS scores as low as 2.0 [3–5]. In addition to physical disability, cognitive impairment is common, with 60–90% of patients with SPMS experiencing cognitive decline [6–8]. Cognitive processing speed is most often affected [9], which in turn also affects higher order cognitive processes. Cognitive dysfunction can predict disability progression as defined by EDSS [10, 11].

From a pathophysiological perspective, RRMS is believed to be driven primarily by peripherally mediated inflammation [3, 5, 12]. The pathophysiology of SPMS is not fully characterized, but it is believed to include chronic inflammation compartmentalized in the central nervous system (CNS) and neurodegeneration associated with the exhaustion of myelin repair mechanisms, leading to neuronal death [3, 5, 12]. Therefore, for a treatment to be effective in patients with SPMS, it needs to target peripheral and central inflammation and neurodegeneration.

Siponimod is an oral, selective sphingosine 1-phosphate (S1P) 1 and 5 receptor modulator. Findings from clinical or preclinical studies support a dual mode of action for siponimod, with peripherally mediated anti-inflammatory effects through modulation of S1P_1_ receptors, resulting in reduced lymphocyte egression from the lymph nodes. This limits the number of circulating lymphocytes that enter the CNS [13, 14]. In preclinical studies, siponimod also exhibited direct anti-inflammatory and remyelination effects through S1P_1_ and S1P_5_ receptors on CNS resident cells [15–19].

The efficacy and safety of siponimod were investigated in EXPAND, a phase 3 study in participants with SPMS, of whom over 50% required walking aids (EDSS ≥ 6.0) at study entry. Siponimod showed superiority over placebo in terms of slowing physical disability progression and cognitive impairment, with significantly greater reductions in annualized relapse rate (ARR), MRI lesion activity and brain volume loss (total and grey matter) and a safety profile similar to that of other S1P receptor modulators [20–23].

Siponimod is approved for the treatment of active SPMS in most countries, or for the treatment of SPMS in some countries [21, 24]. In Europe, siponimod is indicated for the treatment of adult patients with SPMS with active disease evidenced by relapses or imaging features of inflammatory activity [21, 25], while in the USA, the indication is for relapsing forms of MS, to include clinically isolated syndrome, relapsing–remitting disease and active secondary progressive disease [25].

Many labels across the globe indicate siponimod for active SPMS but most available data are for an SPMS population that includes some patients with and some patients without recent signs of disease activity [23]. Thus, there is a need to characterize the efficacy and safety of siponimod specifically in patients with active SPMS to give physicians an understanding of how siponimod acts in these patients. We performed post hoc analyses of data from the subpopulation of participants in EXPAND with active SPMS (defined as presence of relapses in the 2 years before screening and/or at least one T1 gadolinium-enhancing [Gd +] lesion at baseline). The same primary and secondary endpoints reported for the overall population of EXPAND were analysed. In addition, a significantly lower risk of having a clinically meaningful (≥ 4-point) sustained decrease in the Symbol Digit Modalities Test (SDMT) score and a significantly higher likelihood of having a clinically meaningful (≥ 4-point) sustained increase in SDMT score were seen in the overall EXPAND population with siponimod vs placebo [20]. Given the impact that cognitive impairment has on patients with SPMS, we also analysed changes in cognitive processing speed (an exploratory endpoint in EXPAND) in the subgroup of participants with active SPMS.

## Methods

### Standard protocol approvals, registrations and participant consents

The EXPAND study (NCT01665144) adhered to the International Conference on Harmonisation Guidelines for Good Clinical Practice and to the Declaration of Helsinki [26]. The protocol was approved by an independent ethics committee and/or institutional review board at all sites and all participants provided written informed consent before commencing the study, which was funded by Novartis Pharma AG.

## Study design and objectives

The design and primary results of the EXPAND study were reported previously [23]. Briefly, the core part of EXPAND was a double-blind, randomized, placebo-controlled, event- and exposure-driven pivotal phase 3 study of up to 3 years in duration (median duration of exposure: 18 months), investigating the efficacy, safety and tolerability of siponimod in participants with SPMS [23]. Participants between 18 and 60 years of age, with an EDSS score between 3.0 and 6.5 at screening and no relapse history within the previous 3 months were randomized (2:1) to receive once daily oral siponimod 2 mg or placebo. This post hoc subgroup analysis of EXPAND included participants with active SPMS who were randomized and received at least one dose of study drug (full analysis set). Active SPMS was defined as the presence of one or more relapses in the 2 years before screening and/or at least one T1 Gd + lesion at baseline.

## Efficacy outcomes

A list of all prespecified endpoints for the EXPAND study was published [23]. The same primary and secondary endpoints and selected cognition exploratory endpoints prespecified for the EXPAND overall SPMS population were assessed in the subgroup of participants with active SPMS. Primary endpoint: time to 3-month confirmed disability progression (3mCDP), defined as an increase in EDSS score of at least 1.0 if baseline EDSS score was 3.0–5.0, or of at least 0.5 if baseline EDSS score was 5.5–6.5, confirmed after 3 months. Key secondary endpoints: time to 3-month confirmed worsening of at least 20% in the Timed 25-Foot Walk (T25FW), and change from baseline in T2 lesion volume (T2LV) assessed at month 12, at month 24 and averaged over month 12 and month 24. Secondary endpoints: time to 6-month confirmed disability progression (6mCDP; defined as the same as 3mCDP but with changes in EDSS scores confirmed after 6 months), ARR, time to first relapse, change in the 12-item MS Walking Scale (MSWS-12; increasing scores indicate worsening walking ability), change from baseline in percentage brain volume assessed at month 12, at month 24 and averaged over month 12 and month 24, cumulative number of T1 Gd + lesions per MRI scan from post-baseline scans up to and including month 24, percentage of participants with no T1 Gd + lesions on all post-baseline scans in the group of participants with at least one scan post-baseline, number of new or enlarging T2 lesions over all visits, and percentage of participants with no new or enlarging T2 lesions on all post-baseline scans in the group of participants with at least one scan post-baseline. Exploratory endpoint: change in the oral-response version of the SDMT scores [27] (a measure of cognitive processing speed [28]) from baseline to month 24. Exploratory analyses: time to 6-month confirmed clinically meaningful worsening in cognitive processing speed (≥ 4-point decrease in SDMT score), time to 6-month confirmed clinically meaningful improvement in cognitive processing speed (≥ 4-point increase in SDMT score), clinically meaningful worsening/improvement in SDMT scores sustained on all available assessments, and time to 6mCDP and to 6-month confirmed clinically meaningful worsening in cognitive processing speed stratified by previous DMT, including any prior DMT, prior interferon (IFN) at any time or prior IFN as the most recent DMT (exploratory analyses). IFN was the only treatment class with high enough participant numbers to be analysed (*n* = 306 for siponimod vs *n* = 154 for placebo).

MRI brain scans were performed at baseline, 12 months, 24 months, 36 months and at the end of the double-blind core part of the study (if different from annual visits), and were analysed independently at a central reading site (NeuroRx Research, Montreal, QC, Canada) by staff unaware of participant treatment group assignments.

## Safety outcomes

Adverse events and laboratory abnormalities were reported descriptively. Adverse events were coded according to the Medical Dictionary for Regulatory Activities, version 19.0. The percentage of participants with adverse events, the number of adverse events leading to discontinuation and serious adverse events were reported.

## Statistical analysis

Time to 3mCDP and to 6mCDP, time to 3-month confirmed worsening of at least 20% in the T25FW test, time to first confirmed relapse, time to 6-month confirmed worsening/improvement in SDMT scores and sustained worsening/improvement in SDMT scores were analysed using a Cox proportional hazards model. Treatment, country/region, presence of relapses in the 2 years before the study and baseline EDSS score, or baseline T25FW, or baseline number of T1 Gd + lesions, or baseline SDMT score, respectively, were included as covariates for all analyses. For time-to-event data, efficacy was reported as a hazard ratio (HR), quantifying risk reduction with siponimod treatment compared with placebo. Statistical significance was tested at a two-sided 0.05 level. T2 lesion volume (T2LV) and percentage brain volume change were analysed using a mixed model for repeated measures with time as a categorical class variable and an unstructured covariance matrix. Covariates included treatment, country/region, age, baseline T2LV or baseline normalized brain volume, number of T1 Gd + lesions at baseline and presence of relapses in the 2 years before screening. ARR and numbers of lesions (T1 Gd + and T2) were estimated by a negative binomial regression model with treatment, age, baseline EDSS score, baseline number of T1 Gd + lesions or T2 lesions and presence of relapses in the 2 years before screening as covariates. A repeated measures model was used to analyse change from baseline in MSWS-12 scores with visit as a categorical factor and adjustment for treatment, region/country and baseline score. A mixed model for repeated measures was used to analyse change from baseline in SDMT scores with visit as a categorical factor and adjustment for treatment and baseline score. The proportion of participants with clinically meaningful change in SDMT scores (≥ 4 points) were analysed by a Cox regression model adjusted for predictors treatment, country, baseline SDMT score, baseline MS Severity Score and superimposed relapses at baseline; comparisons of categorical proportions were made using the χ^2^ test. Adverse events, adverse events leading to discontinuation and serious adverse events were reported using descriptive statistics. *p* values were not corrected for multiplicity for any of the assessments.

## Results

### Participant demographics and baseline characteristics

In total, 779/1645 participants (47.4%) who entered the EXPAND study (and received at least one dose of study drug) had active SPMS according to the defining criteria (presence of relapses in the 2 years before screening and/or at least one T1 Gd + lesion at baseline) and 621 (79.7%) completed the study. Participant disposition is summarized in Figure S1. To understand the robustness of the active SPMS designation, the enrolled ‘non-active’ group was checked for signs of activity during the study. Of the participants who did not meet the active SPMS definition at enrolment and who were randomized to placebo, 149/283 (52.7%) had evidence of disease activity during the study (of these, 8.7% had relapses, 73.8% had MRI activity and 17.4% had both). Analyses of activity on study in the placebo group of participants who were active at enrolment were also performed. Of the participants who met the active SPMS definition at enrolment and who were randomized to placebo, 19/91 (20.9%) did not have confirmed relapses or MRI activity during the study.

The demographics and baseline characteristics of participants in the active SPMS subgroup and the overall population from the EXPAND study were broadly similar, except for the percentage of participants with relapses in the 2 years before the study and participants with T1 Gd + lesions at baseline, per definition. Participants with active SPMS also had a slightly higher T2 lesion load (12.4 vs 10.0 cm^3^, respectively; Table 1), and tended to be younger with shorter disease durations and time since conversion to SPMS than the overall population. Table S1 provides the demographic and baseline characteristics for the overall populations of participants with active or non-active SPMS.

**Table 1:** Baseline demographics and participant characteristics of the active SPMS subgroup and overall population from the EXPAND study

	All participants with active SPMS		Overall EXPAND population *N* = 1645
	Siponimod *n* = 516	Placebo *n* = 263	
Age, years	46.2 ± 8.1	47.2 ± 8.5	48.0 ± 7.9
Women, *n* (%)	331 (64.1)	166 (63.1)	987 (60.0)
Duration of MS since first symptom, years	15.6 ± 7.9	15.5 ± 8.2	16.8 ± 8.3
Time since conversion to SPMS, years	3.2 ± 3.32	3.1 ± 3.20	3.8 ± 3.5
EDSS score, median (range)	6.0 (2.0, 7.0)	6.0 (2.5, 6.5)	6.0 (2.0, 7.0)
SDMT score	38.1 ± 14.0	38.6 ± 13.2	39.1 ± 13.8
Participants with relapses in the 2 years before screening, *n* (%)	388 (75.2)	202 (76.8)	590 (36.0)
Proportion of participants with T1 Gd + lesions, *n* (%)	236 (45.7)	114 (43.3)	351 (21.3)
T2 lesion volume, cm^3^, median (range)	12.0 (0.0, 116.6)	12.7 (0.0, 103.6)	10.0 (0.0, 116.6)
Normalized brain volume, cm^3^, median (range)	1418 (1171, 1723)	1418 (1228, 1679)	1422 (1136, 1723)

Data are mean ± SD unless otherwise specified

*EDSS* Expanded Disability Status Scale, *Gd* + gadolinium-enhancing, *MS* multiple sclerosis, *SD* standard deviation, *SDMT* Symbol Digit Modalities Test, *SPMS* secondary progressive multiple sclerosis

## Physical disability and relapses

**Fig. 1:**
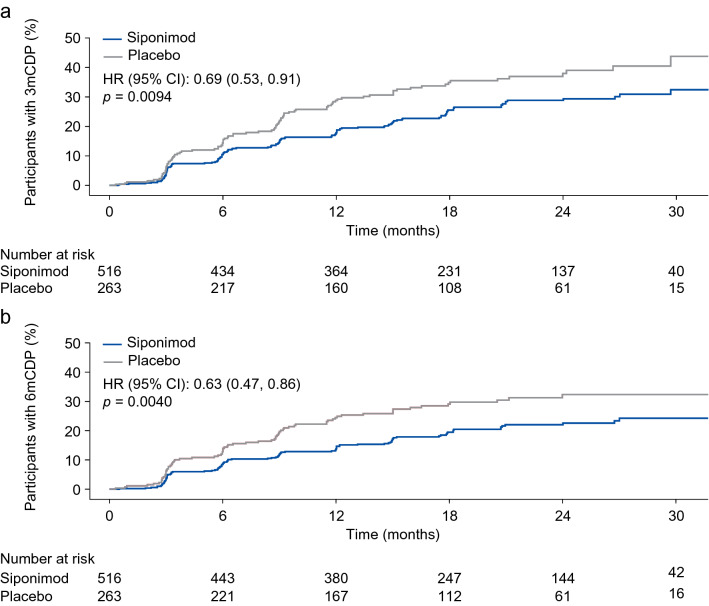
Time to 3mCDP and to 6mCDP in the subgroup of participants from EXPAND with active SPMS. *3mCDP* 3-month confirmed disability progression, *6mCDP* 6-month confirmed disability progression, *CI* confidence interval, *HR* hazard ratio, *SPMS* secondary progressive multiple sclerosis

Siponimod treatment reduced the risk of 3mCDP by 31% compared with placebo (HR: 0.69; 95% confidence interval [CI]: 0.53, 0.91; *p* = 0.0094) (Fig. 1; Table 2).

**Table 2:** Primary and secondary endpoints in participants with active SPMS

	Siponimod (*n* = 516)	Placebo (*n* = 263)	Between-group difference	*p* value
Primary endpoint				
Confirmed disability progression at 3 months, *n*/*N* (%)	128/515^a^ (24.9)	91/263^a^ (34.6)	HR 0.69 (0.53, 0.91)	0.0094
Key secondary endpoints				
*Clinical*				
Worsening of ≥ 20% from baseline in T25FW confirmed at 3 months, *n*/*N* (%)	213/511 (41.7)	120/263 (45.6)	HR 0.85 (0.68, 1.07)	0.1747
*MRI*				
Change from baseline in total volume of lesions on T2-weighted images (mm^3^)				
Month 12, adjusted mean (SE) or [95% CI]	93.485 (129.700)	1117.15 (160.760)	− 1023.7 [− 1355.7, − 691.66]	< 0.0001
Month 24, adjusted mean (SE) or [95% CI]	13.286 (139.710)	1316.32 (175.924)	− 1303.0 [− 1675.8, − 930.31]	< 0.0001
Mean over month 12 and month 24, adjusted mean (SE) or [95% CI]	53.385 (127.514)	1216.73 (156.977)	− 1163.3 [− 1483.9, − 842.78]	< 0.0001
Other secondary endpoints				
*Clinical*				
Confirmed disability progression at 6 months, *n*/*N* (%)	98/515^a^ (19.0)	74/263^a^ (28.1)	HR 0.63 (0.47, 0.86)	0.0040
Annualized relapse rate [95% CI]	0.093 [0.071, 0.121]	0.171 [0.127, 0.230]	RR 45.56 [0.387, 0.766]	0.0005
Change in MSWS-12 score from baseline				
Month 12, adjusted mean (SE) or [95% CI]	1.67 (1.033)	4.17 (1.323)	− 2.50 [− 5.42, 0.42]	0.0926
Month 24, adjusted mean (SE) or [95% CI]	4.48 (1.255)	6.23 (1.632)	− 1.76 [− 5.49, 1.97]	0.3552
Average over all visits, adjusted mean (SE) or [95% CI]	2.54 (0.965)	5.15 (1.202)	− 2.60 [− 5.20, − 0.01]	0.0494
*MRI*				
Change in percentage brain volume loss from baseline				
Month 12, adjusted mean (SE) or [95% CI]	− 0.385 (0.044)	− 0.559 (0.055)	0.173 [0.064, 0.283]	0.0020
Month 24, adjusted mean (SE) or [95% CI]	− 0.861 (0.055)	− 0.969 (0.070)	0.108 [− 0.045, 0.261]	0.1657
Mean over month 12 and month 24, adjusted mean (SE) or [95% CI]	− 0.623 (0.047)	− 0.764 (0.058)	0.141 [0.020, 0.261]	0.0221
Cumulative number of T1 Gd + lesions from baseline up to and including month 24, adjusted mean [95% CI]	0.169 [0.130, 0.219]	1.088 [0.807, 1.467]	RR 0.155 [0.104, 0.231]	< 0.0001
Mean number of new or enlarging T2 lesions on T2-weighted images over all visits, adjusted mean [95% CI]	1.147 [0.911, 1.445]	5.811 [4.811, 7.018]	RR 0.197 [0.149, 0.261]	< 0.0001

*CI* confidence interval, *Gd* + gadolinium-enhancing, *HR* hazard ratio, *MRI* magnetic resonance imaging, *MSWS-12* 12-item Multiple Sclerosis Walking Scale, *RR* rate reduction, *SE* standard error, *SPMS* secondary progressive multiple sclerosis, *T25FW* Timed 25-Foot Walk

^a^Number of subjects with events/number of subjects included in the analysis (i.e., with non-missing covariates)

No significant difference was observed in the time to 3-month confirmed worsening of at least 20% in T25FW (HR: 0.85; 95% CI: 0.68, 1.07; *p* = 0.1747) (Table 2). No significant effects on these endpoints related to physical disability were reported in participants with non-active SPMS (Table S2).

**Fig. 2:**
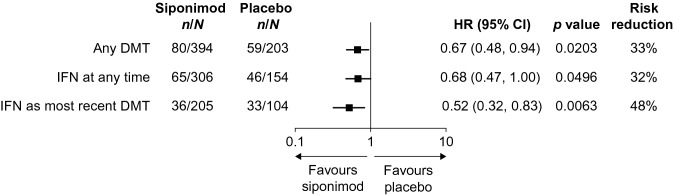
Time to 6mCDP by previous DMT^a^ in the subgroup of participants from EXPAND with active SPMS. *6mCDP* 6-month confirmed disability progression, *CI* confidence interval, *DMT* disease-modifying therapy, *HR* hazard ratio, *IFN* interferon, *MS-DMT* multiple sclerosis-DMT, *SPMS* secondary progressive multiple sclerosis. ^a^Any DMT, participants who received and stopped any MS-DMT before the first dose of siponimod in EXPAND; IFN at any time, participants who received and stopped IFN at any time before the first dose of siponimod in EXPAND; IFN as most recent DMT, participants who received and stopped IFN as most recent MS-DMT before the first dose of siponimod in EXPAND

Siponimod treatment reduced the risk of 6mCDP by 37% (HR: 0.63; 95% CI: 0.47, 0.86; *p* = 0.0040) compared with placebo, an effect that was consistent in participants with any prior DMT at any time (HR: 0.67; 95% CI: 0.48, 0.94; *p* = 0.0203), prior IFN treatment at any time (HR: 0.68; 95% CI: 0.47, 1.00; *p* = 0.0496) and IFN as the most recent treatment (HR: 0.52; 95% CI: 0.32, 0.83; *p* = 0.0063) (Fig. 2).

Change in MSWS-12 scores from baseline (adjusted mean [standard error] averaged over all visits) was 2.54 (0.97) with siponimod and 5.15 (1.20) with placebo (between-group difference: − 2.60; 95% CI: − 5.20, − 0.01; *p* = 0.0494) (Table 2).

ARR was lower in the siponimod-treated group than in the placebo group (0.093 vs 0.171; rate reduction: 0.54; 95% CI: 0.39, 0.77; *p* = 0.0005) as was the time to confirmed first relapse (HR: 0.64, 95% CI: 0.46, 0.89; *p* = 0.0079).

## Cognitive function

Siponimod treatment was associated with benefits in cognitive processing speed, as assessed by SDMT scores (with a change of 4 points or more considered to be clinically meaningful).

**Fig. 3:**
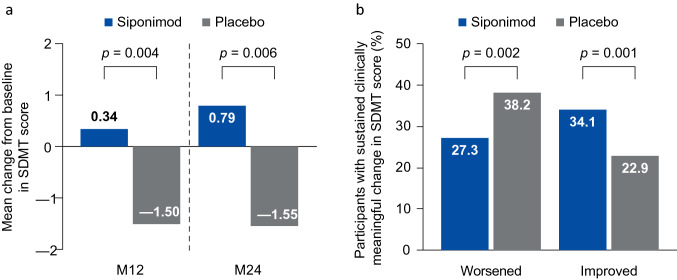
Change in SDMT score from baseline and proportion of participants with sustained clinically meaningful worsening/improvement in SDMT score (≥ 4-point change). *M* month, *SDMT* symbol digit modalities test

**Fig. 4:**
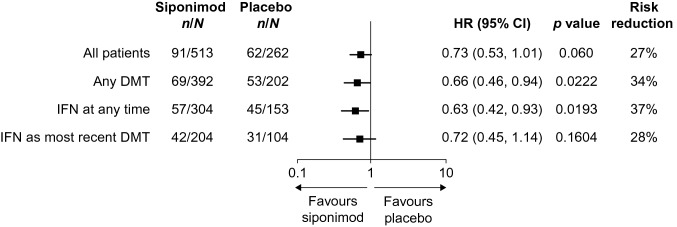
Time to 6-month confirmed worsening in cognitive processing speed (decrease of ≥ 4 points in SDMT score) in the subgroup of participants from EXPAND with active SPMS (all patients and stratified by previous DMT^a^). *CI* confidence interval, *DMT* disease modifying therapy, *HR* hazard ratio, *IFN* interferon, *MS-DMT* multiple sclerosis-DMT, *SDMT* symbol digit modalities test, *SPMS* secondary progressive multiple sclerosis. ^a^Any DMT, participants who received and stopped any MS-DMT before the first dose of siponimod in EXPAND; IFN at any time, participants who received and stopped IFN at any time before the first dose of siponimod in EXPAND; IFN as most recent DMT, participants who received and stopped IFN as most recent MS-DMT before the first dose of siponimod in EXPAND

Mean change in SDMT score from baseline to month 24 was 0.79 with siponimod and − 1.55 with placebo (between-group difference: 2.34; 95% CI: 0.66, 4.02; *p* = 0.006; Fig. 3a). The risk of 6-month confirmed clinically meaningful worsening in cognitive processing speed was numerically reduced (by 27%) with siponimod treatment compared with placebo (HR: 0.73; 95% CI: 0.53, 1.01; *p* = 0.060), an effect that was consistent in participants with any prior DMT treatment (HR: 0.66; 95% CI: 0.46, 0.94; *p* = 0.0222), prior IFN treatment at any time (HR: 0.63; 95% CI: 0.42, 0.93; *p* = 0.0193) and IFN as the most recent treatment (HR: 0.72; 95% CI: 0.45, 1.14; *p* = 0.1604) (Fig. 4). Siponimod significantly decreased the risk of clinically meaningful SDMT worsening sustained over all visits compared with placebo (HR: 0.72; 95% CI: 0.56, 0.94; *p* = 0.0166). The proportions of participants with sustained clinically meaningful worsening in SDMT were 27.3% with siponimod and 38.2% with placebo (*p* = 0.002; Fig. 3b). Siponimod increased the likelihood of 6-month confirmed clinically meaningful SDMT improvement more than placebo, with a likelihood increase of 62% (HR: 1.62; 95% CI: 1.14, 2.29; *p* = 0.007) as well as the likelihood of sustained clinically meaningful improvement (HR: 1.51; 95% CI: 1.12, 2.04; *p* = 0.007). The proportions of participants with sustained clinically meaningful improvement in cognitive processing speed were 34.1% with siponimod and 22.9% with placebo (*p* = 0.001; Fig. 3b). These proportions were not significantly different in participants with non-active SPMS (Table S3).

## MRI outcomes

Siponimod treatment was associated with smaller increases in T2LV and with a reduction in brain volume loss compared with placebo (Table 2). Mean changes in T2LV from baseline to month 24 were 13.3 mm^3^ with siponimod and 1316.3 mm^3^ with placebo (between-group difference: − 1303.0 mm^3^; 95% CI: − 1675.8, − 930.31 mm^3^; *p* < 0.0001). Adjusted mean percentage brain volume change over months 12 and 24 was − 0.62 with siponimod and − 0.76 with placebo (between-group difference: 0.141; 95% CI: 0.020, 0.261; *p* = 0.0221). A significant effect was also observed for participants with non-active SPMS (Table S4).

Siponimod treatment was associated with fewer T1 Gd + lesions and fewer new or enlarging T2 lesions than placebo (relative rates: 0.15; 95% CI: 0.10, 0.22, and 0.20; 95% CI: 0.15, 0.26, respectively; *p* < 0.0001; Table 2). In addition, proportionally more participants receiving siponimod than those receiving placebo were free from T1 Gd + lesions (83.9% vs 54.3%) and from new or enlarging T2 lesions (44.8% vs 25.0%) at all post-baseline scans.

## Safety outcomes

Safety outcomes in participants with active SPMS are summarized in Table S5.

Adverse events were reported in 86.8% of participants with active SPMS receiving siponimod and in 78.3% of those participants receiving placebo. Corresponding proportions for adverse events leading to treatment discontinuation were 5.8% in the siponimod group and 6.1% in the placebo group; for serious adverse events, corresponding proportions were 15.1% in the siponimod group and 15.6% in the placebo group.

## Discussion

In this post hoc analysis of participants with active SPMS, the impact of siponimod on reducing the risk of physical disability progression compared with placebo was more pronounced than that observed in the overall EXPAND population (31% vs 21% risk reduction in 3mCDP and 37% vs 26% risk reduction in 6mCDP, respectively) [23]. Similar to the overall study, an effect of siponimod on T25FW in participants with active SPMS was not observed, possibly owing to the high variability of this measure in a population in which many are already dependent on walking aids, which may have decreased this measure’s sensitivity to change [23]. Safety outcomes in participants with active SPMS were consistent with those observed in the overall population of EXPAND [23].

In addition to physical disability, progressive cognitive impairment in patients with SPMS is a major contributor to overall disability and loss of employment [29]. The SDMT is considered to be the most sensitive performance-based measure of cognitive status in patients with MS and a 4-point change in SDMT is deemed clinically meaningful [28]. In participants with active SPMS, the likelihood of sustained improvement in cognitive processing speed (≥ 4-point increase in SDMT score) was increased by 51% and the risk of sustained worsening (≥ 4-point decrease in SDMT score) was reduced by 28% with siponimod compared with placebo; proportionally more participants with active SPMS experienced sustained improvement (34% vs 23%; *p* = 0.001) and proportionally fewer experienced sustained worsening (27% vs 38%; *p* = 0.002) in cognitive processing speed with siponimod than with placebo. This is consistent with findings in the overall population of EXPAND, in which the likelihood of sustained improvement in cognitive processing speed increased by 28%, the risk of sustained worsening decreased by 21% [20] and the risk of 6-month confirmed worsening decreased by 25% [21]. This suggests that siponimod has the potential to delay or even to reverse cognitive deficits related to processing speed in patients with SPMS. Furthermore, a recent analysis of the EXPAND trial showed that the likelihood of improvement in cognitive processing speed is greater in participants with active SPMS (62%) than in those with non-active disease (19%; Table S3) [30]. These findings suggest that patients with active SPMS have a greater capacity for improvement in cognitive function with siponimod treatment than those with non-active SPMS. However, cognitive worsening is slowed at a similar rate across all patients with SPMS (i.e., those with active and those with non-active SPMS) [20]. It is not immediately apparent why improvements in cognitive processing speed occurred more commonly in participants with active SPMS than in those with non-active SPMS; differences in mean age (46.6 vs 49.5 years in those with active vs non-active SPMS), and duration since first MS symptoms (15.6 vs 18.1 years in those with active vs non-active SPMS; Table S1), and/or other confounders, such as fatigue, depression or level of education, could have played a role. Given that neuronal plasticity and the functional adaptive reserve of the brain decrease with greater age and disease duration [31], it is possible that participants with active SPMS (being younger and/or having shorter disease duration than participants with non-active SPMS) have greater neurological reserve. This greater reserve may, in turn, allow these participants to maximize the effects of siponimod on cognitive processing speed compared with non-active participants (being older and/or having longer disease duration) [20]. Regardless of the explanation, this observation underscores the importance of treating as early as possible in SPMS to preserve and possibly improve cognitive performance.

Consistent with the findings in the overall EXPAND SPMS population, siponimod also reduced inflammatory disease activity as measured by ARR and MRI lesion activity, and the rate of brain volume loss (reaching statistical significance during the first 12 months and on average over months 12 and 24) in the active SPMS subgroup. Further analyses on MRI measures that may provide insights into pathological aspects related to neurodegeneration, such as grey matter atrophy and magnetization transfer ratio (a measure of myelin density), showed pronounced efficacy of siponimod in the overall population (with a consistent effect in participants with active and those with non-active SPMS [32] in line with the significant effects observed on T2LV in both active and non-active SPMS).

Thus, in participants with active SPMS, there was a greater response on clinical outcomes but a similar response on the more sensitive and objective MRI measures related to neurodegeneration and tissue integrity, when compared with participants in the overall or non-active SPMS groups. This suggests that siponimod may work through two (perhaps interlinked) pathophysiological pathways affecting inflammation and neurodegeneration. Initiating siponimod early in patients with active SPMS may present the best window of opportunity to delay physical disability progression, preserve neurological reserve and potentially improve cognitive status.

This post hoc analysis in participants with active SPMS had important limitations. First, the post hoc nature of the study is hypothesis generating and precludes definitive interpretation of the results. Furthermore, the EXPAND study was not powered to assess treatment effects in patients with active and non-active SPMS separately, but rather in the overall EXPAND SPMS population also considering the duration of the core study (median 21 months). Findings from the EXPAND long-term extension study indeed suggest that participants with non-active SPMS progress more slowly than those with active SPMS (based on an approximate 30–50% longer time needed for 6-month confirmed progression on EDSS; Table S6) suggesting that a longer follow-up period than used here would be required to see the full effect of siponimod [33]. However, on the more sensitive objective MRI measures related to neurodegeneration and tissue integrity, consistently significant results were observed in both the active and non-active SPMS subgroups, in line with the reported results in the overall population [32] for the core study. Consistent with these findings, previous analyses have also suggested that the positive effects of siponimod on disability progression may occur independently of relapse activity [34]. Moreover, although the time-to-event design of EXPAND was appropriate for a study in participants with SPMS, further timepoint comparisons in subgroups were complicated by the variable study duration and the fact that switching participants with confirmed disability progression from receiving placebo to receiving open-label siponimod was allowed.

In conclusion, the beneficial treatment effects of siponimod on clinical outcomes during the core study duration were more obvious in participants with active SPMS than in the overall EXPAND population. This is possibly as a result of the combined impact on peripheral anti-inflammatory effects as well as central effects of siponimod, a more responsive population who are slightly younger with likely still higher reserve capacity [31, 35] and potentially the sensitivity of the different clinical endpoints to assess meaningful changes over a given time period. These data combined with a safety profile that was consistent between participants with active SPMS and the overall EXPAND population (and consistent with that of S1P modulation) support the value of siponimod for the treatment of patients with SPMS.

## Supplementary Information

Below is the link to the electronic supplementary material.

Supplementary file1 (DOCX 106 KB)

## Data Availability

Novartis is committed to sharing, with qualified external researchers, access to patient-level data and supporting clinical documents from eligible studies. These requests are reviewed and approved by an independent review panel on the basis of scientific merit. All data provided are anonymized to respect the privacy of patients who have participated in the trial, in line with applicable laws and regulations. This trial data availability is according to the criteria and process described on https://www.clinicalstudydatarequest.com/.
