# Supplementary material for: Siponimod vs placebo in active secondary progressive multiple sclerosis: a post hoc analysis from the phase 3 EXPAND study
Source: J Neurol. 2022 May 31;269(9):5093–104. doi: 10.1007/s00415-022-11166-z (PMC9363350; doi:10.1007/s00415-022-11166-z)
Supplement: Supplementary file 1 — Supplementary file1 (DOCX 106 KB) [file 415_2022_11166_MOESM1_ESM.docx]

**Supplementary information**

**Table S1** Baseline demographics and participant characteristics of the overall active and non-active SPMS subgroups

|  | All participants with active SPMS (*N* = 779) | All participants with non‑active SPMS (*N* = 827) |
| --- | --- | --- |
| Age, years | 46.6 ± 8.3 | 49.5 ± 7.2 |
| Women, *n* (%) | 497 (63.8) | 470 (56.8) |
| Duration of MS since first symptom, years | 15.6 ± 8.0 | 18.1 ± 8.5 |
| Time since conversion to SPMS, years | 3.2 ± 3.3 | 4.3 ± 3.6 |
| EDSS score, median (range) | 6.0 (2.0, 7.0) | 6.0 (2.5, 7.0) |
| SDMT score | 38.3 ± 13.7 | 40.0 ± 13.7 |
| Participants with relapses in the 2 years before screening, *n* (%) | 590 (75.8) | 0 (0.0) |
| Proportion of participants with T1 Gd+ lesions, *n* (%) | 350 (45.9) | 0 (0.0) |
| T2 lesion volume, cm^3^, median (range) | 12.4 (0.0, 116.7) | 8.6 (0.0, 99.0) |
| Normalized brain volume, cm^3^, median (range) | 1418 (1171, 1723) | 1426 (1136, 1691) |

Data are mean ± SD unless otherwise specified

*EDSS* Expanded Disability Status Scale, *Gd+* gadolinium-enhancing, *MS* multiple sclerosis, *SD* standard deviation, *SDMT* Symbol Digit Modalities Test, *SPMS* secondary progressive multiple sclerosis

**Table S2** Primary and secondary endpoints in participants with non-active SPMS

|  | Siponimod  (*n*= 557) | Placebo  (*n*= 270) | Between-group difference | *p* value |
| --- | --- | --- | --- | --- |
| Primary endpoint | | | | |
| Confirmed disability progression at 3 months, *n*/*N* (%) | 153/557^a^ (27.5) | 78/270^a^ (28.9) | HR 0.93  (0.71, 1.23) | 0.6209 |
| Key secondary endpoints | | | | |
| *Clinical* | | | | |
| Worsening of ≥ 20% from baseline in T25FW confirmed at 3 months, *n*/*N* (%) | 210/552 (38.0) | 101/268 (37.7) | HR 1.00  (0.79, 1.28) | 0.9711 |
| *MRI* | | | | |
| Change from baseline in total volume of lesions on T2-weighted images (mm³) | | | | |
| Month 12, adjusted mean (SE) or [95% CI] | 244.3 (80.89) | 482.3 (100.35) | −238.0 [−432.1, −43.8] | 0.0164 |
| Month 24, adjusted mean (SE) or [95% CI] | 247.8 (85.11) | 505.5 (107.07) | −257.7  [−472.4, −42.9] | 0.0188 |
| Mean over month 12 and month 24, adjusted mean (SE) or [95% CI] | 246.1 (79.97) | 493.9 (98.56) | −247.8  [−437.3, −58.3] | 0.0105 |
| Other secondary endpoints | | | | |
| *Clinical* | | | | |
| Confirmed disability progression at 6 months, *n*/*N* (%) | 114/557^a^ (20.5) | 62/270^a^ (23.0) | HR 0.87  (0.64, 1.19) | 0.3763 |

*CI* confidence interval, *HR* hazard ratio, *MRI* magnetic resonance imaging, *SE* standard error, *SPMS* secondary progressive multiple sclerosis, *T25FW* Timed 25-Foot Walk

^a^Number of subjects with events/number of subjects included in the analysis (i.e., with non-missing covariates)

**Table S3** Summary of cognitive endpoint analysis for participants with non-active SPMS

|  | Siponimod  (*n* = 551) | Placebo (*n* = 266) | Between-group difference (95% CI) | *p* value |
| --- | --- | --- | --- | --- |
| Mean change in SDMT score from baseline to month 24 | 1.92 | −0.53 | 2.44 (0.67, 4.22) | 0.007 |
| Proportion of patients with sustained^a^ clinically meaningful change in SDMT score during core study |  |  |  |  |
| Worsened, *n* (%) | 117 (21.2) | 63 (23.7) |  | 0.429 |
| Improved, *n* (%) | 196 (35.6) | 83 (31.2) |  | 0.217 |
| 6m confirmed clinically meaningful^b^ worsening in SDMT |  |  | HR 0.76  (0.53, 1.09) | 0.140 |
| 6m confirmed clinically meaningful^b^ improvement in SDMT |  |  | HR 1.19  (0.86, 1.65) | 0.300 |

*6m* 6-month, *CI* confidence interval, *HR* hazard ratio, *SDMT* Symbol Digit Modalities Test, *SPMS*, secondary progressive multiple sclerosis

^a^≥4-point change in the SDMT score from baseline that continued until the end of the follow up in the core part without ever returning to above or below this threshold

^b^≥4-point change in the SDMT score

**Table S4** Summary of exploratory MRI endpoint analyses [[32](#_ENREF_32)]; PPS^a^

|  | Siponimod | Placebo | *p* value |
| --- | --- | --- | --- |
| Percentage change in total brain volume from baseline to month 24 |  |  |  |
| Active SPMS (siponimod *n* = 350; placebo *n* = 173) | −0.81 | −1.03 | 0.0138 |
| Non-active SPMS (siponimod *n* = 344; placebo *n* = 167) | −0.40 | −0.75 | 0.0005 |
| Percentage change in cGM volume from baseline to  month 24 |  |  |  |
| Active SPMS (siponimod *n* = 347; placebo *n* = 169) | −0.50 | −1.14 | < 0.0001 |
| Non-active SPMS (siponimod *n* = 344; placebo *n* = 167) | −0.27 | −0.94 | < 0.0001 |
| Percentage change in thalamic volume from baseline to month 24 |  |  |  |
| Active SPMS (siponimod *n* = 348; placebo *n* = 173) | −1.41 | −2.15 | 0.0032 |
| Non-active SPMS (siponimod *n* = 347; placebo *n* = 168) | −0.56 | −1.34 | 0.0009 |

*cGM* cortical grey matter, *MRI* magnetic resonance imaging, *PPS* per-protocol set, *SPMS* secondary progressive multiple sclerosis

^a^ PPS included all patients from the full analysis set who did not have any major protocol deviations that could confound interpretation.

**Table S5** Overview of adverse events in the active SPMS subgroup

|  | **Siponimod  (*N =* 516)** | **Placebo  (*N =* 263)** |
| --- | --- | --- |
| **Event** | | |
| Any adverse event | 448 (86.8) | 206 (78.3) |
| Adverse event leading to discontinuation of study drug | 30 (5.8) | 16 (6.1) |
| Death^a^ | 2 (0.4) | 1 (0.4) |
| Any serious adverse event | 78 (15.1) | 41 (15.6) |
| **Areas of interest with S1P receptor modulators** | | |
| Liver-related investigations, signs and symptoms (SMQ broad) | 72 (14.0) | 12 (4.6) |
| Alanine aminotransferase increased (PT) | 34 (6.6) | 6 (2.3) |
| Gamma-glutamyl transferase increased (PT) | 24 (4.7) | 2 (0.8) |
| Hypertension (SMQ narrow) | 66 (12.8) | 24 (9.1) |
| Hypertension (PT) | 54 (10.5) | 19 (7.2) |
| Thromboembolic events (NMQ) | 14 (2.7) | 6 (2.3) |
| Infections and infestations (SOC) | 238 (46.1) | 117 (44.5) |
| Herpes zoster/shingles (NMQ) | 11 (2.1) | 1 (0.4) |
| Herpes simplex virus infections (NMQ) | 13 (2.5) | 5 (1.9) |
| Skin neoplasms, malignant and unspecified (SMQ narrow) | 3 (0.6) | 4 (1.5) |
| Basal cell carcinoma (PT) | 2 (0.4) | 4 (1.5) |
| Lymphopenia (PT) | 4 (0.8) | 0 (0.0) |
| Lymphocyte count decreased (PT) | 2 (0.4) | 0 (0.0) |
| Oedema peripheral (PT) | 19 (3.7) | 3 (1.1) |
| Macular oedema (PT) | 7 (1.4) | 1 (0.4) |
| Convulsions, including all types of seizure (SMQ broad) | 5 (1.0) | 2 (0.8) |
| Bradyarrhythmias, including conduction defects, during treatment initiation (NMQ) | 48 (9.3) | 9 (3.4) |
| Bradycardia (PT) | 31 (6.0) | 8 (3.0) |
| Sinus bradycardia (PT) | 10 (1.9) | 1 (0.4) |
| Conduction defects (SMQ broad) | 9 (1.7) | 0 (0.0) |
| **Serious adverse events occurring in ≥ 0.5% of patients in either group** | | |
| Alanine aminotransferase increased | 6 (1.2) | 2 (0.8) |
| Anaemia | 0 (0.0) | 2 (0.8) |
| Aspartate aminotransferase increased | 3 (0.6) | 1 (0.4) |
| Basal cell carcinoma | 2 (0.4) | 3 (1.1) |
| Gait disturbance | 0 (0.0) | 2 (0.8) |
| Laceration | 4 (0.8) | 0 (0.0) |
| Suicide attempt | 3 (0.6) | 2 (0.8) |
| Syncope | 3 (0.6) | 1 (0.4) |
| Urinary tract infection | 6 (1.2) | 2 (0.8) |

Data are number of patients (%)

*MedDRA* Medical Dictionary for Regulatory Activities, *MS* multiple sclerosis, *MS-DMT* multiple sclerosis disease-modifying therapy, *NMQ* Novartis MedDRA query, *PT* preferred term, *S1P* sphingosine 1‑phosphate, *SMQ* standardized MedDRA query, *SOC* system organ class, *SPMS* secondary progressive MS

^a^In the siponimod group, causes of death were suicide and urosepsis (after the patient started an alternative MS-DMT). Both deaths were considered unrelated to treatment. In the placebo group, the cause of death was lung adenocarcinoma

**Table S6** Summary of extension study clinical findings [[33](#_ENREF_33)]

| Outcome | Placebo-siponimod | Continuous siponimod | HR (95% CI) |
| --- | --- | --- | --- |
| 6-month confirmed disability progression percentiles (months) |  |  |  |
| Active SPMS |  |  | 0.71 (0.57, 0.90), *p*= 0.0044 |
| 25^th^ | 12.0 | 21.3 |  |
| 40^th^ | 28.1 | 43.5 |  |
| Non-active SPMS |  |  | 0.88 (0.69, 1.11),  *p* = ns |
| *25^th^* | 15.4 | 21.0 |  |
| *40^th^* | 41.3 | 44.9 |  |

*CI* confidence interval, *HR* hazard ratio*, ns* not significant*, SPMS* secondary progressive multiple sclerosis

**Fig. S1** Participant disposition for the active SPMS subgroup (all randomized patients)


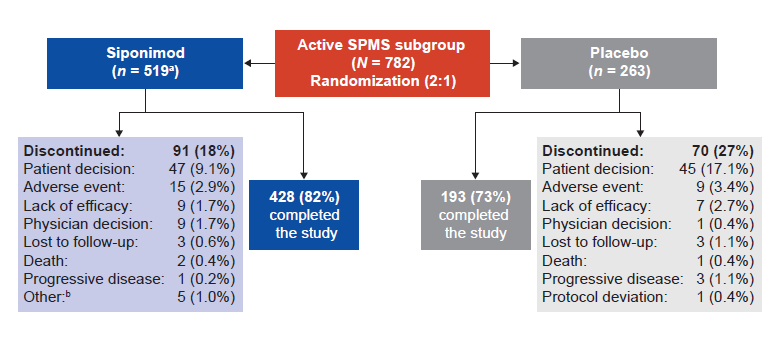


*SPMS* secondary progressive multiple sclerosis

^a^Three patients randomized to the siponimod arm did not receive treatment

^b^Treatment non-compliance (*n =* 2), protocol deviation (*n =* 1), technical problems (*n =* 1), and new therapy for the study indication (*n =* 1)
